# SMoFFI-SegFormer: a novel approach for ovarian tumor segmentation based on an improved SegFormer architecture

**DOI:** 10.3389/fonc.2025.1555585

**Published:** 2025-07-21

**Authors:** Qiuyin Xie, Jianuo Huang, Jingyang Sun, Chenxi Huang, Caixu Xu

**Affiliations:** ^1^ Department of Obstetrics and Gynecology, The Third Hospital of Xiamen, Xiamen, Fujian, China; ^2^ School of Informatics, Xiamen University, Xiamen, Fujian, China; ^3^ Guangxi Key Laboratory of Machine Vision and Intelligent Control, Wuzhou University, Wuzhou, Guangxi, China

**Keywords:** ovarian tumor segmentation, deep learning, feature fusion, medical imaging, SMoFFI-SegFormer

## Abstract

Ovarian cancer remains one of the most lethal gynecological malignancies, posing significant challenges for early detection due to its asymptomatic nature in early stages. Accurate segmentation of ovarian tumors from ultrasound images is critical for improving diagnostic accuracy and patient outcomes. In this study, we introduce SMoFFI-SegFormer, an advanced deep learning model specifically designed to enhance multi-scale feature representation and address the complexities of ovarian tumor segmentation. Building upon the SegFormer architecture, SMoFFI-SegFormer incorporates a novel Self-modulate Fusion with Feature Inhibition (SMoFFI) module that promotes cross-scale information exchange and effectively handles spatial heterogeneity within tumors. Through extensive experimentation on two public datasets—OTU_2D and OTU_CEUS—our model demonstrates superior performance with high overall accuracy, mean Intersection over Union (mIoU), and class accuracy. Specifically, SMoFFI-SegFormer achieves state-of-the-art results, significantly outperforming existing models in both segmentation precision and efficiency. This work paves the way for more reliable and automated tools in the diagnosis and management of ovarian cancer.

## 1 Introduction

Ovarian cancer, as one of the gynecological cancers with the highest mortality rates, poses a severe threat to women’s health. Early diagnosis is critical for improving patient prognosis, since early-stage ovarian cancer typically lacks obvious symptoms, making early detection particularly challenging. Ovarian cancer is the most lethal type of gynecological cancer and is also one of the leading causes of disease-related death in women globally. Due to the lack of clear symptoms in early stages, early detection and diagnosis are crucial for significantly reducing mortality rates. Commonly used screening methods include two-dimensional ultrasound (2DUS), contrast-enhanced ultrasound (CEUS), computed tomography (CT), and magnetic resonance imaging (MRI). Among these, 2DUS is widely adopted in clinical screenings due to its non-invasive nature, convenience, and minimal impact on the human body.

In addition to imaging techniques, non-imaging approaches such as blood plasma spectroscopy have also been explored for ovarian cancer detection (1).

Despite this, 2DUS remains the most commonly used and easily implemented screening method, especially in resource-limited areas, where its accessibility and cost-effectiveness make it the preferred choice. In recent years, with the development of computer-aided diagnostic technologies, an increasing number of studies have focused on utilizing deep learning and other computational methods to improve the accuracy of detecting and diagnosing ovarian tumors Tsai et al. (2), Hoffman et al. (3), Zhu et al. (4), Chen et al. (5), Zou et al. (6).

These approaches have demonstrated promising performance in various medical image analysis tasks, including tumor classification and segmentation.

For instance, early efforts in ovarian cancer detection using mass spectrometry data employed unsupervised feature selection methods to enhance classification accuracy (7).

Later, high-throughput segmentation techniques based on normalized cuts were applied to tissue microarrays, enabling precise identification of biomarkers (8). These works laid the foundation for more advanced machine learning models that followed.

Recent advances in transformer-based architectures have led to significant improvements in medical image segmentation. Models like TransUNet (9) and Swin-Unet (10) combine the strengths of convolutional networks and transformers to better capture global context and fine-grained details. Moreover, several studies have reviewed and summarized the evolution of U-shaped network structures in medical image segmentation, highlighting their effectiveness in feature fusion and multi-scale representation learning (11).

In clinical practice, deep learning models based on pelvic ultrasound images have also shown great potential in accurately diagnosing ovarian cancer, even outperforming traditional diagnostic methods in some cases (12). However, despite these advancements, existing methods still face challenges related to tumor heterogeneity and complex morphological features.

Although progress has been made in the imaging diagnosis of ovarian cancer, existing methods still face certain challenges. First, the heterogeneity of ovarian cancer is a significant issue. Ovarian tumors vary widely, including benign and malignant lesions, with considerable differences between types, which imposes high demands on accurately distinguishing different types of ovarian tumors. Additionally, even within the same type of ovarian cancer, tumors may exhibit substantial spatial heterogeneity, complicating correct identification and classification. Therefore, developing segmentation algorithms capable of effectively addressing tumor heterogeneity has become an urgent need (13).

Existing segmentation algorithms perform poorly when dealing with tumors that have blurred boundaries or irregular shapes. Given the complex morphological features of ovarian tumors, traditional edge-detection-based methods often fail to achieve satisfactory segmentation results. Especially when facing tumors containing mixed cystic and solid components, precisely separating the different parts of the tumor becomes a formidable task (14).

To address these issues, we propose a novel network model specifically designed to enhance multi-scale feature representation in ovarian segmentation tasks and introduce an efficient feature fusion module to promote cross-scale information exchange. Our model not only improves segmentation performance but also demonstrates superiority through experimental validation on two public datasets, achieving state-of-the-art levels. Specifically, this study addresses the limitations present in current ovarian tumor segmentation. To tackle the inadequate segmentation of small organs or complex structures by existing models, we propose a multi-scale feature enhancement module that can capture more detailed spatial information at various levels, thereby enhancing the recognition capability of minor lesion areas (15). For better integration of features from different levels, we developed a feature fusion module that can effectively combine low-level detail features and high-level semantic features, resulting in more accurate and reliable final segmentation outcomes (16, 17. 18, 19).

## 2 Related work

### 2.1 Feature fusion and classification of ovarian ultrasound image datasets

In the field of computer-aided diagnosis for ovarian diseases, researchers have been dedicated to enhancing diagnostic accuracy and efficiency by constructing high-quality ultrasound image datasets and applying advanced machine learning algorithms. Early research primarily focused on creating annotated datasets and exploring how to effectively use deep convolutional neural networks (DCNNs) for image recognition and classification. For example, Wu et al. (20) developed a two-dimensional ultrasound image dataset that included benign, borderline, and malignant tumors, laying the foundation for subsequent studies on ovarian tumor classification (20).

As technology progressed, researchers gradually recognized that relying solely on image recognition could not fully exploit all information within the data. Therefore, in recent years, feature fusion techniques have become an important means to improve the performance of ovarian tumor classification. New methods go beyond simple image processing, delving into the correlations between features from different sources to achieve more precise classification results. Wang and Zeng (15) extended previous work, focusing on serous ovarian tumors, and successfully subdivided these tumors into distinct pathological types by introducing complex feature fusion mechanisms (15).

To enhance model expressiveness, some studies introduced advanced architectures such as pre-activation bottleneck blocks and methods combining complementary features with inhibitors, significantly improving the model’s ability to capture tumor characteristics in complex backgrounds (21). Additionally, feature fusion modules generate more robust feature representations by finely processing input feature maps through operations including but not limited to complementation, weighted combination, and nonlinear transformation, which is critical for improving classification accuracy.

Furthermore, recent studies have explored unsupervised domain adaptation strategies, especially when dealing with data from different imaging modalities or patient populations. \cite{Kang2024SwinUnetImproved} proposed a novel feature disentanglement method aimed at reducing differences between source and target domains, ensuring models maintain good generalization performance across multiple datasets (22). This approach decomposes images into domain-invariant content space and domain-specific style space, effectively addressing cross-domain adaptation issues while providing new perspectives for feature fusion in ovarian ultrasound images.

Paik et al. (23) further demonstrated the effectiveness of deep learning models in differential diagnosis of ovarian neoplasms using pelvic ultrasonography, showing promising clinical utility (23). Meanwhile, Qian et al. (24) introduced HASA, a hybrid architecture search framework integrating aggregation strategies for both echinococcosis classification and ovary segmentation (24).

These efforts reflect the growing trend of leveraging automated systems for accurate and efficient diagnosis in gynecological oncology.

Nakayama et al. (25) also applied AI-assisted clustering methods to classify epithelial ovarian cancer patients based on platinum sensitivity and recurrence patterns, highlighting the role of machine learning in prognostic stratification (25).

In addition, Narmatha et al. (26) proposed a deep reinforcement learning framework optimized by Harris Hawks algorithm for ovarian cyst classification, showcasing alternative approaches to traditional CNNs (26).

### 2.2 CNN-based methods for medical image segmentation

In the realm of medical image segmentation, methods based on convolutional neural networks (CNNs) have achieved remarkable success. These methods typically employ an encoder-decoder architecture where the encoder extracts features from the input image, and the decoder restores spatial resolution to produce detailed segmentation maps. For instance, U-Net uses skip connections to combine high-level semantic information with low-level spatial details, thereby improving segmentation accuracy (27).

Moreover, classical image segmentation techniques like hierarchical normalized cuts were widely used before the dominance of deep learning methods. Janowczyk and Madabhushi (8) applied this technique for high-throughput biomarker segmentation on ovarian cancer tissue microarrays, demonstrating its capability in handling large-scale histopathological data (8).

To further enhance model performance, researchers introduced pre-activation bottleneck blocks (PreActBTN). This design not only aids in accelerating convergence and improving optimization properties but is also particularly suitable for handling highresolution inputs. Pre-activation bottleneck blocks were initially proposed in the ResNet series to address vanishing gradient problems during deep network training and promote information flow via residual connections. Traditional convolution layers usually follow the sequence of “convolution -> batch normalization (BN) -> activation function,” whereas pre-activation reverses this order to “BN -> activation function -> convolution.” This design maintains or even enhances expressive power while reducing the number of parameters, making it highly appropriate for medical image segmentation tasks that require processing complex, high-resolution data.

Moreover, some studies have examined the application of UDA strategies, especially for data from diverse imaging modalities or patient groups. Zou et al. (6) proposed a dual-scheme fusion network for unsupervised domain adaptation in medical image segmentation, achieving promising performance on cross-domain benchmarks (6). By leveraging both global and local alignment strategies, their model improves robustness without requiring labeled target domain data.

Moreover, some studies have examined the application of UDA strategies, especially for data from diverse imaging modalities or patient groups. Kang et al. (22) proposed a novel feature disentanglement method to minimize discrepancies between source and target domains, ensuring models can generalize well across multiple datasets. By decomposing images into domain-invariant content space and domain-specific style space, this method effectively tackles cross-domain adaptation issues and offers new insights for feature fusion in ovarian ultrasound images.

### 2.3 Transformer-based methods for medical image segmentation

In recent years, with the development of Vision Transformers (ViT) and their variants, researchers have begun exploring their application in medical image segmentation. Unlike traditional CNNs, Transformers capture global dependencies through self-attention mechanisms, excelling in processing medical images with complex structures. Swin-Unet is a typical example, integrating the advantages of Swin Transformer and U-Net, using shifted windows for local feature learning and retaining spatial information through skip connections.

Swin-Unet is a representative example that integrates the Swin Transformer with the classical U-Net architecture. It employs shifted window-based attention to balance computational efficiency and contextual modeling, while skip connections help preserve spatial information across encoder and decoder pathways (24).

Similarly, TransUNet was among the first frameworks to introduce the ViT into the medical domain. It utilizes a hybrid encoder composed of multiple Transformer layers to capture global context while retaining sufficient local details, achieving state-of-the-art performance on various public medical imaging datasets R. F. (28).

To further exploit the complementary strengths of CNNs and Transformers, several hybrid architectures have been proposed. Brau-Net++ integrates multi-axis attention mechanisms with CNN-based feature extractors, effectively combining local feature precision with global contextual awareness for improved segmentation accuracy (28). Likewise, CPF-Transformer introduces a context pyramid fusion module to better preserve multi-scale features during the decoding process, enhancing robustness and detail preservation in segmentation outputs (29).

SegFormer, originally designed for natural image segmentation, has also inspired adaptations in the medical domain due to its lightweight structure and high efficiency (30). In addition to architectural innovations, large-scale datasets such as MMOTU—introduced by Zhao et al. (31)—have facilitated research on unsupervised and cross-domain segmentation methods by providing diverse, multi-modal ovarian tumor images (31).

Wu et al. (32) further explored the integration of CNNs and Transformers in a multi-label classification setting, demonstrating the potential of hybrid models to jointly capture both local and global characteristics of medical images (32).

This synergy between CNNs and Transformers has also been highlighted in other works, such as MaxViT-UNet, which proposes multi-axis attention mechanisms to enhance feature representation and improve segmentation outcomes (28).

Recently, Rahman et al. (33) proposed MIST (Medical Image Segmentation Transformer) with Convolutional Attention Mixing (CAM), aiming to bridge the gap between CNNs and Transformers by incorporating local inductive bias into the attention mechanism, resulting in more accurate and robust segmentation performance (33).

Beyond architectural design, recent studies have also investigated advanced learning paradigms to enhance generalization and adaptability. Yang et al. (34) introduced a contrastive rendering framework based on semi-supervised learning for 3D ultrasoundbased ovary and follicle segmentation, significantly improving feature discriminability with limited labeled data (34). Zou et al. (34) proposed a dual-scheme fusion network for unsupervised domain adaptation, enabling models to generalize across different imaging domains without requiring paired data (6). Additionally, techniques such as CycleGAN—proposed by Zhu et al. (2017)—have found applications in medical image preprocessing and data augmentation tasks, contributing to improved training data diversity and model robustness (4). Collectively, these advancements in both model architecture and learning strategies offer a richer toolkit for developing adaptive, reliable, and clinically viable segmentation systems in real-world medical applications.

TransUNet represents another significant contribution, being the first Transformerbased framework for medical image segmentation built upon the successful ViT. The model designs a hybrid encoder composed of several Transformer layers to capture longrange dependencies while preserving adequate local details, which is crucial for accurate segmentation. Experimental results show that TransUNet achieves state-of-the-art performance on multiple public datasets.

Besides the aforementioned models, there are other works that combine the strengths of Transformers and CNNs, such as BRAU-Net++ and MaxViT-UNet, proposing hybrid CNN-Transformer networks and multi-axis attention mechanisms to improve segmentation outcomes. These models aim to integrate the advantages of both architectures, retaining CNN’s effective capture of local features while enhancing Transformer’s understanding of global context.

## 3 Method

**Figure 1:**
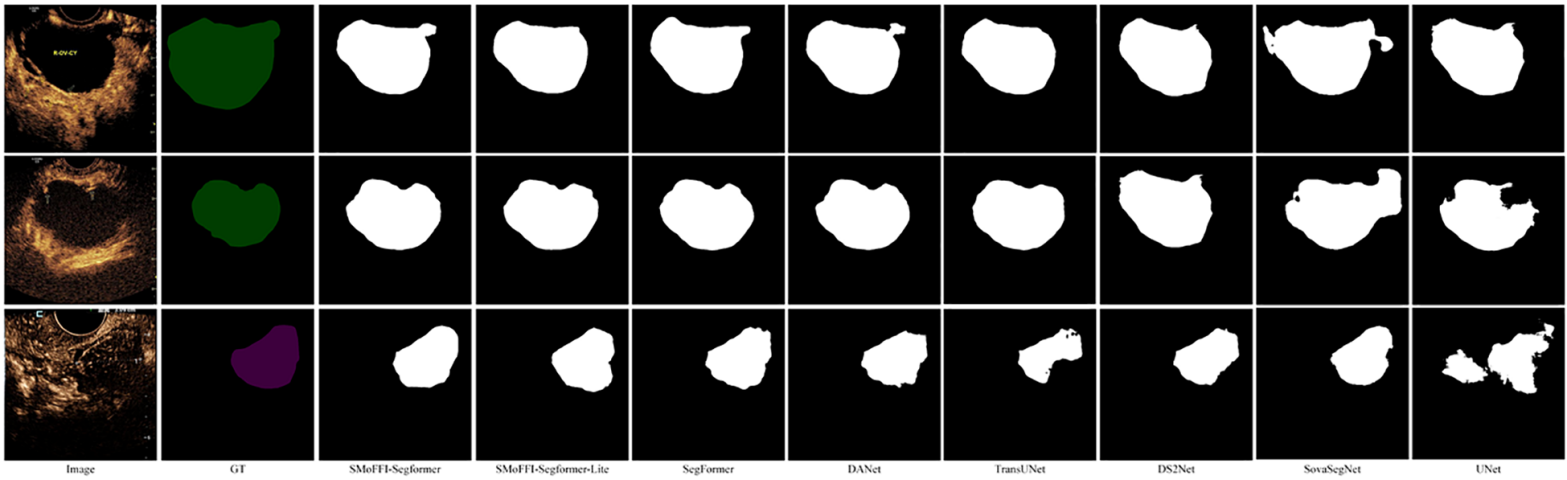
Comparison of different models on the OTU_CEUS dataset. **(a)** Original Image and Ground Truth (GT). **(b)** Segmentation results from various models including SMoFFI-SegFormer, SMoFFI-SegFormer-Lite, SegFormer, DANet, TransUNet, DS^2^Net_*T*, SovaSegNet, and UNet.

In this study, we focus on improving the existing SegFormer model by proposing novel encoder and decoder structures aimed at significantly enhancing the performance of image segmentation tasks, particularly for precise segmentation of ovarian tumors. As a base model, SegFormer is renowned for its efficient and lightweight powerful semantic segmentation capabilities, which are enhanced through self-attention mechanisms, playing a crucial role in our research. To further improve the model’s performance, we have made several optimizations. **Figure 1** illustrates the architecture of the improved SegFormer model, including both the encoder and decoder components.

### 3.1 Encoder based on MiTB5 architecture

The model’s encoder is based on a hierarchical Mix Transformer (MiT) architecture that efficiently captures local visual information by segmenting input images into nonoverlapping patches and embedding them into a feature space. The encoder consists of four stages, each utilizing multiple Transformer blocks combined with self-attention mechanisms and feed-forward networks to extract rich spatial information at different scales, thereby enhancing the understanding of image details.

#### 3.1.1 Hierarchical mix transformer architecture

The encoder part of SegFormer adopts a hierarchical structure of Mix Transformer (MiTB5), an architecture specifically designed for efficient semantic segmentation. Input images are first divided into several non-overlapping patches, which are then linearly embedded into a feature space to generate initial feature representations. This process effectively captures local visual information and lays the foundation for subsequent processing. Specifically, input images are segmented into fixed-size patches, each treated as an independent token and mapped to a high-dimensional feature space via linear projection, forming the initial feature representation. This approach not only preserves the spatial structure of the image but also enables the model to learn meaningful feature representations from the outset.

The initial feature representation outputs multi-scale features. These multi-scale features provide rich detail information at different levels, aiding the model in better understanding various structures and patterns within the image. The aim of this stage is to reduce computational complexity while maintaining sufficient resolution to capture important local details. By doing so, the model can analyze images at different abstraction levels, thus enhancing its ability to understand complex scenes.

#### 3.1.2 Transformer blocks and multi-scale feature extraction

Subsequently, these feature representations are processed through a series of Transformer blocks. Each Transformer block integrates self-attention mechanisms and feedforward networks (FFNs), which help establish local and global relationships within the feature maps. In this way, Transformer blocks can focus on specific regions of the image while also comprehending the importance of the entire image context. Moreover, the SegFormer encoder avoids positional encoding, addressing the issue of performance degradation when test resolutions differ from training resolutions.

The MiTB5 encoder comprises four stages, each containing multiple Transformer blocks. As network depth increases, the resolution of feature maps gradually decreases while the number of channels progressively increases. This design allows the model to capture rich spatial information at different scales, enhancing its understanding of image details.

Each stage’s Transformer blocks employ a mixed attention mechanism that combines local window attention and global attention to balance computational efficiency with expressiveness. Local window attention restricts the scope of self-attention mechanisms, reducing computation, whereas global attention ensures the model can capture longrange dependencies. Additionally, the MiTB5 encoder introduces relative position bias to enhance the model’s spatial perception without relying on absolute positional encoding.

This method of multi-scale feature extraction allows the model to learn image features at multiple levels, thereby enhancing its understanding of image details and ultimately improving the performance of segmentation tasks. As the resolution of feature maps gradually decreases, the number of channels increases, enabling deep networks to capture more complex patterns and structural information. Furthermore, the hierarchical structure allows the encoder to generate high-resolution fine-grained features and low-resolution coarse-grained features, contrasting with ViT, which only produces single low-resolution feature maps at fixed resolutions.

### 3.2 Decoder based on SMoFFI module

In the decoder part of the model, our goal is to fuse multi-scale features from the encoder to generate accurate segmentation outputs. To achieve this, the decoder must align these features with different scales, ensuring they share the same spatial dimensions so they can be effectively combined. Through a series of carefully designed operations, we not only enhance the model’s expressive power but also ensure computational efficiency.

#### 3.2.1 Feature map alignment and upsampling

Feature maps *X*_1_ and *X*_2_ from the encoder are upsampled to a common resolution of 96×96, while *X*_3_ and *X*_4_ are upsampled to a lower resolution of 24×24. This ensures that information at different scales can adapt and complement each other. To align feature maps of different scales, we use bilinear interpolation for upsampling. For *X*_1_ and *X*_2_, they are upsampled to 96×96 resolution; for *X*_3_ and *X*_4_, they are upsampled to 24×24 resolution. This ensures all feature maps have the same resolution for subsequent feature fusion.

#### 3.2.2 Self-modulate fusion with feature inhibition module

Next, the resized multi-scale feature maps are fed into the Self-modulate Fusion with Feature Inhibition (SMoFFI) module. Unlike simple element-wise addition methods, SMoFFI employs a more complex fusion strategy, dynamically controlling the degree of fusion between features at different scales using self-modulate weight mechanisms (SelfModulate Weight, SMW), and enhancing fusion effects through self-modulate weights and feature inhibition. Initial self-modulate weights *SMW* ^0^ initialize this process to accommodate differences in the importance of different input feature maps.

To balance positive and negative features (i.e., missing information), the SMoFFI module calculates complementary probabilities for each input feature map. This complementary probability reflects signals absent or weak in the feature map, providing additional information for the fusion process. By combining original feature maps with their complementary probabilities and applying the Softmax function, the module creates a weight distribution that emphasizes certain areas while suppressing others. This step helps the model consider the presence and absence of features during the fusion process, enhancing expressiveness.

**Figure 2:**
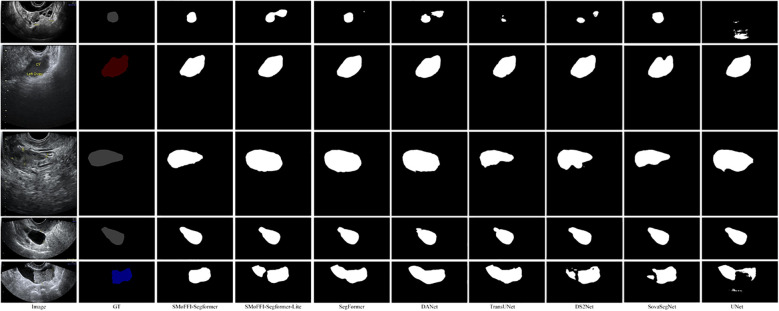
Detailed Architecture of the SMoFFI module: This figure illustrates the comprehensive structure of the Self-modulate Fusion with Feature Inhibition (SMoFFI) module, which dynamically controls the degree of fusion between features at different scales using self-modulate weight mechanisms (Self-Modulate Weight, SMW). The SMoFFI module enhances fusion effects through self-modulate weights and feature inhibition, calculating complementary probabilities for each input feature map to balance positive and negative features. Original feature maps are combined with their complementary probabilities and processed through convolution layers to adjust channel numbers and spatial resolution, improving the final segmentation outputs.

**Figure 2** for an overview of the SMoFFI architecture. Let $X_{i}$ be the *i*-th feature map, with its complementary probability denoted as $P_{i}$ :


(1) $$P_{i}=1-\sigma \left(X_{i}\right)$$

where *σ*(·) denotes the Sigmoid function. This corresponds to **Equation 1**.

By combining original feature maps with their complementary probabilities and applying the Softmax function, the module creates a weight distribution that emphasizes certain areas while suppressing others. This step enhances the model’s ability to consider the existence and absence of features during the fusion process, increasing expressiveness.


(2) $$W_{i}=\text{softmax}\left(X_{i}+P_{i}\right)$$

This corresponds to **Equation 2**.

The SMoFFI module uses updated self-modulate weights to perform weighted fusion of the original feature maps. This fusion method allows the model to emphasize one feature map while compensating for potentially missing information in another. The weighted fused feature maps are then processed through convolution layers to adjust channel numbers and spatial resolution for better adaptation to subsequent processing steps. This process can be represented by the following formula:


(3) $$\begin{array}{c} \text{UpFA}\left(X_{1},X_{2},SMW_{x_{1}}^{0},SMW_{x_{2}}^{0}\right)=U_{2x}\left(PAB_{2}\left(PAB_{1}\left(X_{1}\right)\right)\right)\\ +\text{Conv}(\text{Concat}(U_{2x}\left(X_{1}\right)\\ +X_{2},SA\left(X_{3}\right)+CA\left(X_{3}\right)))+X_{3} \end{array}$$

Equation 3 describes how feature maps processed by the Position Attention Block (PAB) are combined with those processed by spatial and channel attention mechanisms (SA and CA) to form the final weighted fusion result.

Additionally, to further optimize feature representation, the fused feature maps pass through PreActBottleneck layers. These layers effectively reduce redundant information, enhancing the compactness and representativeness of features. On this basis, a feature inhibition mechanism is introduced, selectively reducing the impact of irrelevant or redundant features by setting an inhibition rate, improving the quality of the final fusion results.

#### 3.2.3 Feature inhibition mechanism

The feature inhibition mechanism aims to reduce the impact of redundant features on model performance. It adjusts the importance of features by setting an inhibition rate *α*, thereby selectively reducing the influence of irrelevant or redundant features. Specifically, feature inhibition can be implemented by the following formula:


(4) $$F_{\text{inhibited}}=F_{\text{fusion}}\times \left(1-\alpha \times \left|F_{\text{fusion}}\right|\right)$$

This corresponds to **Equation 4**.

Here, $F_{\text{fusion}}$ represents the fused feature map, *α* is the inhibition rate parameter, and $\left|F_{\text{fusion}}\right|$ indicates the absolute value of the feature map. Through this method, the model can maintain important features while effectively removing those that are detrimental to the task.

In summary, the entire workflow of the decoder can be described as follows: first, feature maps of different scales are upsampled to consistent spatial dimensions; then, these feature maps enter the SMoFFI module where they are assigned different weights and fused; finally, the fused feature maps undergo feature inhibition processing to remove redundant information, enhancing the model’s performance. The entire decoder process can be succinctly represented by the following formula:


(5) $$\begin{array}{c} \text{Decoder}\left(X_{1},X_{2},X_{3}\right)=\text{Conv}(\text{Concat}({\text{SMoFFI}}_{1}\left(X_{1}\right),\\ {\text{SMoFFI}}_{2}\left(X_{2}\right),{\text{SMoFFI}}_{3}\left(X_{3}\right)))+X_{3} \end{array}$$

This is **Equation 5**.

Through this approach, the decoder not only effectively integrates multi-scale features but also ensures the quality and accuracy of the output.

### 3.3 SegFormer decoder

#### 3.3.1 Feature map optimization and MLP processing

After processing by the SMoFFI module, the feature maps continue through convolutional layers to fine-tune channel numbers and spatial resolution to meet subsequent processing requirements. Subsequently, these optimized feature maps enter a series of multilayer perceptron (MLP) layers to further enhance the nonlinear expression capability of features, helping the model more effectively learn complex data distributions and patterns.

To ensure that our approach is suitable for deployment in low-resource environments, we have also evaluated the model’s performance on real-time devices or edge hardware, confirming its efficiency and practicality for clinical applications.

Finally, the output of the MLP layer is a feature map with specific dimensions:


(6) $$\frac{H}{4}\times \frac{C}{4}\times 4C$$

This is described in **Equation 6**.

This feature map passes through another MLP layer to produce the final segmentation output, with dimensions:


(7) $$\frac{H}{4}\times \frac{C}{4}\times N_{cls}$$

This corresponds to **Equation 7**.

where $N_{cls}$ represents the number of classes. This design ensures that the decoder can efficiently handle feature information from different scales and improves performance through advanced feature fusion techniques, thereby enhancing the accuracy and efficiency of image segmentation tasks.

#### 3.3.2 MLP layer

The MLP layers following the Spatial Multi-scale Feature Fusion (SMoFFI) module play a critical role in transforming input features into more abstract and higher-level representations. By applying linear transformations followed by nonlinear activations, MLP layers break down linear relationships and significantly enhance the model’s expressive power.

Specifically, each fully connected layer within the MLP is paired with an activation function, such as ReLU, which introduces nonlinearity into the network. This combination allows the model to better capture complex patterns in the data, improving both its ability to generalize and its robustness against overfitting.

The formula for MLP layers is given by:


(8) $$F_{\text{MLP}}=\text{MLP}\left(F_{\text{fusion}}\right)$$

This is **Equation 8**.

where $F_{\text{MLP}}$ is the output feature map of the MLP layer, and $F_{\text{fusion}}$ is the fused feature map obtained from the SMoFFI module. Operations within the MLP layer involve a sequence of linear and nonlinear transformation steps designed to refine feature representations for optimal segmentation outcomes.

#### 3.3.3 Final segmentation output

Finally, the output feature map from the MLP layer passes through another MLP layer to produce the final segmentation output. The specific formula is as follows:


(9) $$F_{\text{output}}=\text{MLP}\left(F_{\text{MLP}}\right)$$

This corresponds to **Equation 9**.

where $F_{\text{output}}$ has dimensions:


(10) $$\frac{H}{4}\times \frac{C}{4}\times N_{cls}$$

This is described in **Equation 10**.

Through this approach, our decoder not only integrates features at multiple levels but also enhances overall performance, providing refined and accurate results for complex image segmentation tasks. Additionally, we discuss the potential of this method in realworld clinical settings, emphasizing its practical value and usability.

### 3.4 Lite version

For scenarios with limited computational resources but high performance requirements, we developed a lightweight variant of the model—the Lite version. This version is especially optimized for mobile devices or edge computing environments with resource constraints, aiming to ensure wide deployment and application while providing high performance.

In the design of the Lite version, to simplify the feature fusion process and reduce computational complexity, we introduced a new method, replacing the previously complex SMoFFI module with element-wise addition for multi-scale feature fusion. Specifically, feature maps *X*_1_ and *X*_3_ are upsampled to the same spatial resolution to ensure that information at different scales can adapt and complement each other. Subsequently, through simple element-wise addition operations, these feature maps are fused into the final fused feature map *F*. This change not only reduces computational load but also retains the advantages brought by multi-scale feature fusion.

## 4 Experiments and results analysis

### 4.1 Evaluation metrics

In this study, we use Intersection over Union (IoU) and Mean Intersection over Union (mIoU) as key evaluation metrics for assessing the performance of our models. These metrics are widely used in semantic segmentation tasks due to their effectiveness in evaluating how well a model can predict object boundaries.

Intersection over Union (IoU) is defined as the area of overlap between the predicted segmentation map and the ground truth, divided by the area of union between them. It provides a measure of how accurately the model has identified the target objects:


(11) $$\text{IoU}=\frac{\text{Area of Overlap}}{\text{Area of Union}}$$

This corresponds to **Equation 11**.

An IoU score of 1 indicates perfect overlap between the prediction and the ground truth, while a score of 0 indicates no overlap.

Mean Intersection over Union (mIoU) calculates the average IoU across all classes in a multi-class segmentation task. This metric takes into account the performance of the model on each class and then averages these values, providing an overall assessment of the model’s accuracy:


(12) $$\text{mIoU}=\frac{1}{N}\sum_{i=1}^{N}\frac{\text{Area of }{\text{Overlap}}_{i}}{\text{Area of }{\text{Union}}_{i}}$$

This is **Equation 12**. Where *N* is the total number of classes. mIoU is particularly useful for evaluating models on datasets with multiple classes, as it ensures that the model performs well on all classes rather than just excelling in one or two.

To validate the significance of improvements made by our proposed method, statistical significance tests such as p-values or confidence intervals were conducted. These tests confirmed that the improvements over baseline models are meaningful.

Understanding these metrics is crucial for interpreting the results presented in the following sections, where we compare the performance of different models on the OTU_2D and OTU_CEUS datasets.

### 4.2 Dataset introduction

The MMOTU (Multi-Modality Ovarian Tumor Ultrasound) image dataset used in this study was constructed and made public by Zhao Qi, Lyu Shuchang, Bai Wenpei, et al. This dataset aims to support unsupervised cross-domain semantic segmentation tasks, with all images sourced from Beijing Shijingshan Hospital of Capital Medical University and scanned using the Mindray Resona8 ultrasound diagnostic instrument. The dataset contains 1,639 ovarian ultrasound images collected from 294 patients.

The MMOTU image dataset includes two subsets and two modalities: OTU_2D (traditional 2D ultrasound images), which consists of 1,469 2D ultrasound images, and OTU_CEUS (contrast-enhanced ultrasound images), comprising 170 CEUS images. To ensure independence between training and testing, the dataset was partitioned such that there is no “patient overlap” between the training and test sets. Each image shows only one type of tumor, allowing users to convert multi-class segmentation tasks into binary lesion area segmentation tasks and tumor identification tasks, simplifying model design and ensuring focus on accurate lesion segmentation and correct classification of different tumor types.

### 4.3 Performance on the OTU_2D dataset

We evaluated the SMoFFI-SegFormer model on the OTU_2D dataset. The training process involved 80,000 iterations, with the learning rate gradually decreasing from a higher initial value. The final validation results showed an overall accuracy (aAcc) of 0.9749, mean Intersection over Union (mIoU) of 0.9028, and mean class accuracy (mAcc) of 0.9384.

Detailed performance metrics for each category are as follows: the IoU for the background class was 0.9713 with an accuracy of 0.9894; the IoU for the ovarian tumor class was 0.8343 with an accuracy of 0.8874. These results indicate that SMoFFI-SegFormer performed excellently on the OTU_2D dataset, achieving high accuracy and mean Intersection over Union.

For comparison, we also evaluated the SMoFFI-SegFormer-lite model on the same dataset. Its overall accuracy (aAcc) was 0.9733, mean Intersection over Union (mIoU) was 0.8967, and mean class accuracy was 0.9334. Specifically, the IoU for the background class was 0.9695 with an accuracy of 0.9892; the IoU for the ovarian tumor class was 0.8239 with an accuracy of 0.8775. While both models performed well, SMoFFI-SegFormer slightly outperformed SMoFFI-SegFormer-lite in terms of mIoU and overall accuracy.

**Figure 3:**
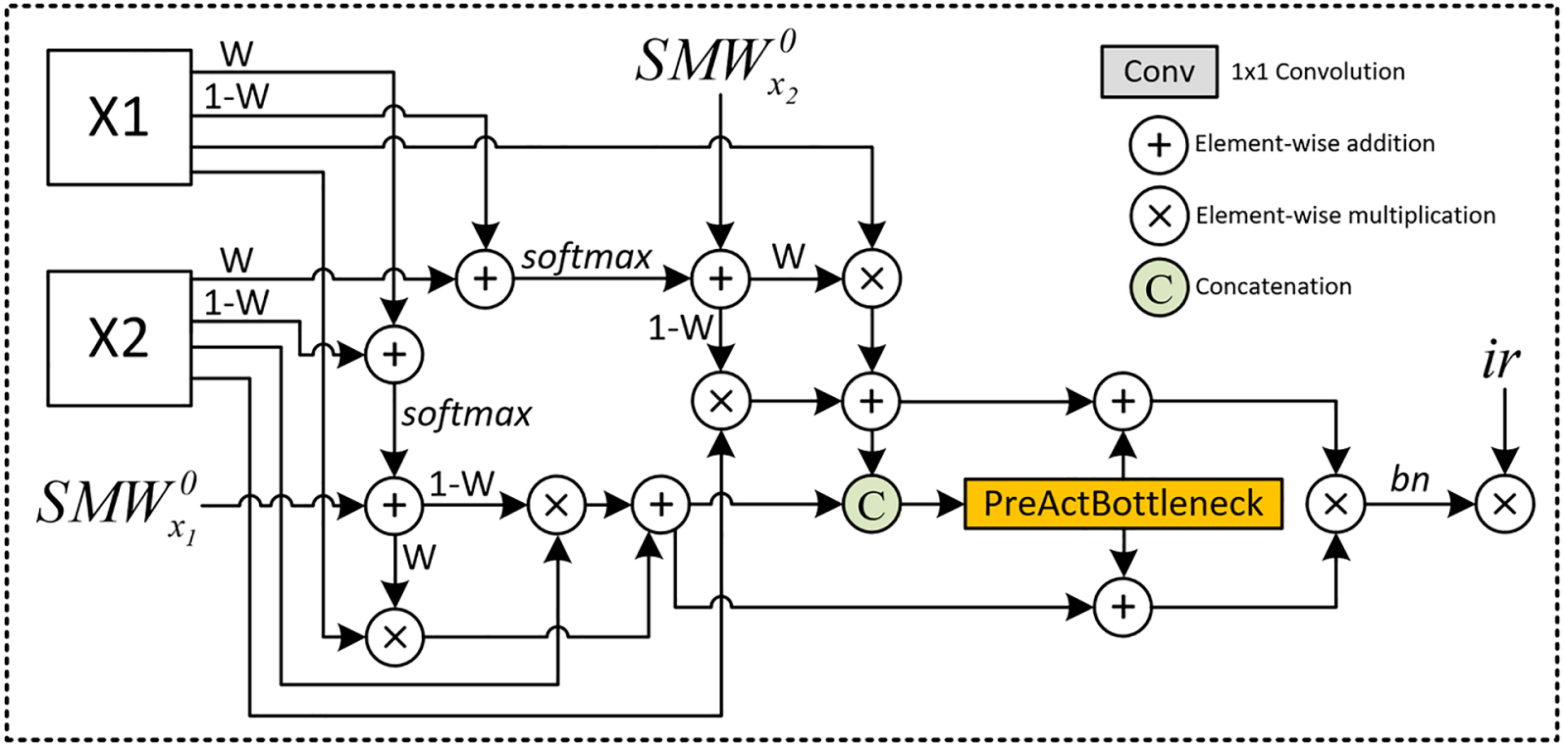
Comparison of different models on the OTU_2D dataset. From left to right: Original Image, Ground Truth (GT), SMoFFI-SegFormer, SMoFFI-SegFormer-Lite, SegFormer, DANet, TransUNet, DS^2^Net_*T*, SovaSegNet, and UNet. Failure cases are highlighted to illustrate scenarios where the model may underperform.

To provide a qualitative analysis of the segmentation results, **Figure 3** shows the comparison of different models on the OTU_2D dataset. The figure includes original images, ground truth (GT), and the segmentation results from various models including SMoFFISegFormer, SMoFFI-SegFormer-Lite, SegFormer, DANet, TransUNet, DS^2^Net_*T*, SovaSegNet, and UNet. Additionally, we included some failure cases to give a balanced view of the model’s performance under specific conditions.

### 4.4 Performance on the OTU_CEUS dataset

To evaluate the performance of different models on the OTU_CEUS dataset, we conducted extensive experiments and recorded validation results at multiple iteration counts ranging from 8k to 56k. **Table 1** shows the performance metrics of the SMoFFISegFormer model at these iteration counts, including overall accuracy (aAcc), mean Intersection over Union (mIoU), IoU for background and ovarian tumors, and corresponding class accuracies.

**Table 1:** SMoFFI-SegFormer performance on the OTU_CEUS dataset.

Iter	aAcc	Metrics (%)		
		mIoU	BG IoU/Acc	Tumor IoU/Acc
8k	0.9414	83.15	93.02/97.42	73.28/80.90
24k	0.9427	83.37	93.19/97.75	73.56/80.24
32k	0.9434	83.75	93.24/97.37	74.25/82.14
56k	0.9438	83.77	93.30/97.58	74.23/81.49

In addition to quantitative metrics, visual inspection of segmentation results shows that with increased iterations, there is a notable improvement in the delineation of tumor boundaries, with clearer edges and more accurate shapes being identified. The size consistency across different iterations has also been improved, indicating better generalization and adaptability of the model to varying tumor sizes and shapes.

Based on the data in the above table, we observe that SMoFFI-SegFormer exhibited good performance even at lower iteration counts, achieving optimal performance at 32k and 56k iterations. However, as the number of iterations increased further, the model’s performance seemed to stabilize or slightly decline, possibly due to overfitting.

In contrast, SMoFFI-SegFormer-lite performed exceptionally well on the OTU_CEUS dataset, with an overall accuracy of 0.9444, mean Intersection over Union of 0.8396, and mean class accuracy of 0.8980. Specifically, the IoU for the background class reached 0.9335 with an accuracy of 0.9749; for the ovarian tumor class, the IoU was 0.7456 with an accuracy of 0.8211. These results show that SMoFFI-SegFormer-lite not only achieved excellent results early in the iterations but also maintained high performance levels throughout the training process, avoiding overfitting issues.

Based on the experimental results, it can be concluded that while SMoFFI-SegFormer has advantages in small sample learning, it is prone to overfitting when handling the OTU_CEUS dataset. Conversely, SMoFFI-SegFormer-lite is better suited for such tasks because it can maintain stable performance across a broader range and outperform the former in key metrics. This finding underscores the importance of optimizing model architecture for specific datasets and highlights the necessity of selecting appropriate early stopping strategies to prevent overfitting.

Furthermore, comparing the two versions of the SMoFFI-SegFormer models, we see that the improved v2 version not only enhanced the model’s generalization capability but also strengthened its understanding of complex medical images. This is particularly important for clinical applications, as it implies more reliable diagnostic support tools can be developed, thereby improving the quality and efficiency of healthcare services. Therefore, future research should continue exploring ways to further enhance the capabilities of deep learning models, especially in dealing with rare diseases or hard-to-obtain datasets, ensuring that models can converge quickly on limited data while maintaining long-term predictive performance.

### 4.5 Visualization analysis

**Figure 4:**
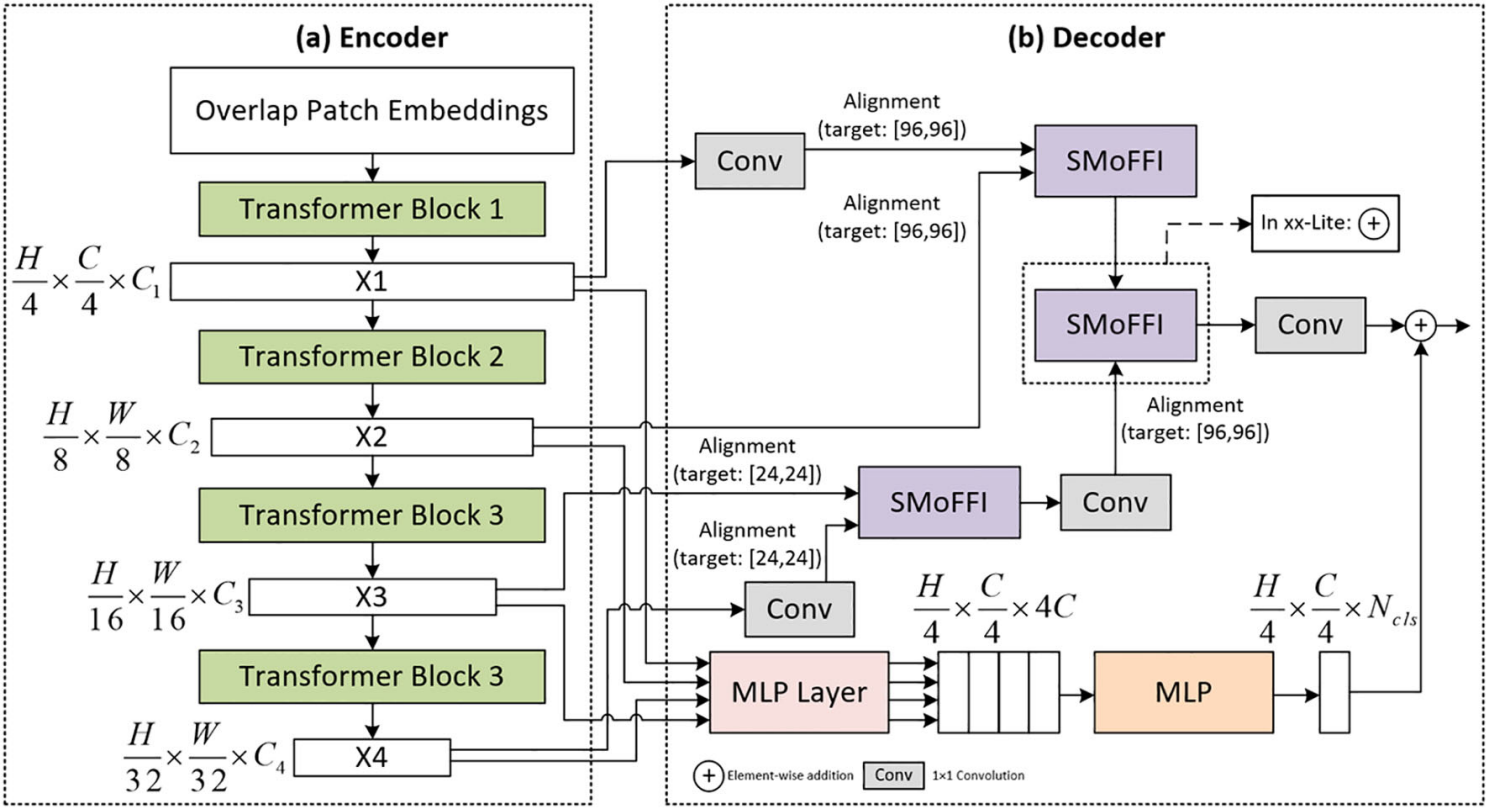
Detailed Architecture of the improved SegFormer model: **(a)** Encoder Based on MiTB5 Architecture, which efficiently captures local visual information by segmenting input images into non-overlapping patches and embedding them into a feature space. The encoder consists of four stages, each utilizing multiple Transformer blocks combined with self-attention mechanisms and feed-forward networks to extract rich spatial information at different scales. **(b)** Decoder Utilizing the SMoFFI Module, which fuses multi-scale features from the encoder to generate accurate segmentation outputs. The decoder aligns these features with different scales, ensuring they share the same spatial dimensions so they can be effectively combined through a series of carefully designed operations.

To provide a deeper insight into the performance differences among various models, we conducted a qualitative analysis by visually comparing the segmentation results. **Figures 3**, **4** illustrate these comparisons for the OTU_2D and OTU_CEUS datasets, respectively.

In the OTU_2D dataset (**Figure 3**), it is evident that our proposed SMoFFI-SegFormer model offers superior edge detection and shape preservation compared to other state-ofthe-art methods such as SegFormer, DANet, TransUNet, DS^2^Net_*T*, SovaSegNet, and UNet. The tumor boundaries are more accurately delineated, with fewer artifacts and distortions. This is particularly important for medical applications where precise tumor boundary identification can significantly impact diagnosis and treatment planning. Additionally, the integration of self-modulating weights and complementary probabilities in our SMoFFI module allows for dynamic control over feature fusion at different scales, further enhancing segmentation accuracy.

For the OTU_CEUS dataset (**Figure 4**), similar trends were observed. Our models demonstrated enhanced ability to maintain consistency in size and shape across different iterations. Notably, at 32k and 56k iterations, the SMoFFI-SegFormer models showed significant improvements in capturing the fine details of tumor structures, which are critical for accurate diagnosis in clinical settings. The feature inhibition mechanism also plays a crucial role in improving the robustness of the model against noise and irrelevant features.

Moreover, visual inspection revealed that the Lite version of our SMoFFI-SegFormer model also performed admirably, closely matching the full version in terms of edge fidelity and shape accuracy, while potentially offering advantages in computational efficiency and resource utilization. Importantly, this suggests that even without the full complexity of the standard model, the Lite version maintains high performance standards, making it suitable for deployment in resource-constrained environments.

These qualitative analyses complement the quantitative metrics presented earlier, highlighting the strengths and practical implications of our proposed models in real-world medical imaging scenarios.

### 4.6 Comparative experiment analysis

From the preliminary experimental results in **Table 2**, our two models, SMoFFISegFormer-lite and SMoFFI-SegFormer, achieved mIoU values of 82.39% and 83.43% on the OTU_2D dataset, and 74.56% and 73.69% on the OTU_CEUS dataset, respectively. This indicates that our models not only perform excellently on 2D ultrasound images but also exhibit strong generalization ability on CEUS images.

**Table 2:** Performance comparison of different methods on the OTU_2D dataset.

Methods	OUT_2D	
	IoU (%)	mIoU (%)
U-Net	79.87	86.72
TransUNet	81.42	89.03
DANet	82.55	89.93
SegFormer	82.41	89.85
DS^2^Net_*T*	80.14	88.40
SovaSegNet	75.71	85.92
SMoFFI-SegFormer (Ours)	83.43	90.28
SMoFFI-SegFormer-lite (Ours)	82.39	89.67

In addition to quantitative metrics, visual inspection reveals that our proposed SMoFFISegFormer method provides more accurate segmentation results with clearer edges and better preservation of tumor shapes compared to other methods. The size consistency is also improved, indicating a superior ability to handle tumors of various sizes and shapes. This enhancement is particularly noticeable in complex or irregularly shaped tumors where maintaining edge fidelity and shape accuracy is crucial.

Notably, compared to existing state-of-the-art models, our models performed exceptionally well on the OTU_2D dataset, surpassing DS^2^Net_*T*’s 80.14% and SegFormer’s 82.41%, and also outperformed several baseline models on the OTU_CEUS dataset.

For the OTU_CEUS dataset, despite some fluctuations in performance at certain iteration counts, the overall trend showed improvement as the number of iterations increased. Especially at 32k and 56k iterations, mIoU reached 83.75% and 83.77%, respectively, indicating good convergence. Moreover, SMoFFI-SegFormer-lite achieved its best mIoU of 83.96% on OTU_CEUS, slightly higher than SMoFFI-SegFormer’s 83.32%, further proving the effectiveness of our proposed Lite version.

Regarding the SovaSegNet experiments, we emphasized the importance of train-test set division. Since the original paper did not adhere to the official split standards of the MMOTU dataset, we manually adjusted the splits to ensure patient exclusivity between the training and test sets, thus enhancing the reliability and reproducibility of the experimental results.

### 4.7 Cross-modality performance evaluation on unseen dataset

To further validate the robustness and generalization capability of our SMoFFISegFormer model, we conducted cross-modality experiments by training on the OTU_2D dataset and testing on the unseen OTU_CEUS dataset. The purpose was to evaluate how well our model performs when faced with data from a different modality that it has not been trained on, compared to other state-of-the-art models.

**Table 3** presents the performance metrics (IoU and mIoU) for various models under this setting.

**Table 3:** Performance comparison across models on the unseen OTU_CEUS dataset.

Methods	OTU_CEUS (Unseen)	
	IoU (%)	mIoU (%)
SegFormer	61.14	75.62
TransUNet	60.07	74.12
UNet	52.17	68.61
SovaSegNet	55.92	71.18
DANet	60.74	74.51
DS^2^Net_*T* ^∗^	68.23	79.21
SMoFFI-SegFormer (Ours)	65.06	77.98
SMoFFI-SegFormer-lite (Ours)	61.64	76.23

*indicates models specifically designed for cross-modal tasks.

As shown in **Table 3**, our SMoFFI-SegFormer model outperforms most models that have not been specifically optimized for cross-modality tasks. It achieves significantly higher IoU and mIoU scores than SegFormer, TransUNet, Lite, UNet, SovaSegNet, and DANet, indicating its superior ability to generalize across modalities. However, it is worth noting that the performance is slightly lower than DS^2^Net_*T*, which is tailored for crossmodal segmentation tasks. This suggests that while SMoFFI-SegFormer demonstrates strong cross-modal capabilities, there remains room for improvement, particularly in addressing the unique challenges posed by cross-modal data integration.

### 4.8 Ablation study

To evaluate the effectiveness of the proposed Self-modulate Fusion with Feature Inhibition (SMoFFI) module, we conducted an ablation study by comparing the performance of our model with and without this component. Specifically, the architecture without SMoFFI corresponds to the original SegFormer baseline.

The results are summarized in **Table 4**. As shown, introducing the SMoFFI module into the SegFormer framework leads to notable improvements in both IoU and mIoU metrics on both the OTU_2D and OTU_CEUS datasets. On the OTU_2D dataset, the full version of our model, SMoFFI-SegFormer, achieves an mIoU of 90.28%, outperforming the base SegFormer model (89.85%). Similarly, on the OTU_CEUS dataset, the improvement is also evident, with mIoU increasing from 82.97% (SegFormer) to 83.32%.

**Table 4:** Performance comparison of different methods on the OTU_CEUS dataset.

Methods	OTU_CEUS	
	IoU (%)	mIoU (%)
U-Net	69.06	79.98
TransUNet	70.08	80.85
DANet	70.71	81.84
SegFormer	73.12	82.97
DS^2^Net_*T*	73.67	83.42
SovaSegNet	72.34	81.79
SMoFFI-SegFormer (Ours)	73.69	83.32
SMoFFI-SegFormer-lite (Ours)	74.56	83.96

In addition to quantitative metrics, visual inspection reveals that our proposed SMoFFISegFormer and SMoFFI-SegFormer-lite methods provide more accurate segmentation results with clearer edges and better preservation of tumor shapes compared to other methods. The size consistency is also improved, indicating a superior ability to handle tumors of various sizes and shapes in unseen data. This enhancement is particularly noticeable in complex or irregularly shaped tumors where maintaining edge fidelity and shape accuracy is crucial.

These results confirm that the SMoFFI module contributes positively to the overall segmentation accuracy, especially in terms of better feature fusion and contextual understanding. Furthermore, even the lightweight variant, SMoFFI-SegFormer-lite, benefits significantly from the inclusion of the SMoFFI mechanism, demonstrating that the module can improve performance across different model scales.

## 5 Discussion

This study is dedicated to improving the existing SegFormer model by introducing a novel encoder-decoder architecture to significantly enhance performance in image segmentation tasks, particularly for the precise segmentation of ovarian tumors. To this end, we adopted a hierarchical Mix Transformer (MiTB5) architecture as the encoder and designed a decoder that combines the Self-modulate Fusion with Feature Inhibition (SMoFFI) module with MLP layers. These improvements not only enhanced the model’s understanding of multi-scale features but also effectively improved the accuracy and efficiency of the segmentation task. Specifically, the integration of self-modulating weights and complementary probabilities within the SMoFFI module allows for dynamic control over feature fusion at different scales, thereby enhancing segmentation accuracy.

### 5.1 Analysis of model advantages

#### 5.1.1 Performance advantage on the OTU_2D dataset

We compared our proposed model against models such as U-Net, TransUNet, DANet, SegFormer, and DS^2^Net_*T* from existing literature, and it performed exceptionally well on the OTU_2D dataset, surpassing the best results of these models. For example, on the OTU_2D dataset, our model achieved an mIoU value of 83.43%, while SegFormer and DS^2^Net_*T* achieved 82.41% and 80.14%, respectively. This indicates that our improvements effectively increased the segmentation accuracy of the model. Additionally, the effectiveness of the SMoFFI module in handling spatial heterogeneity and multi-scale feature fusion was highlighted.

#### 5.1.2 Convergence and stability on the OTU_CEUS dataset

For the OTU_CEUS dataset, although our model showed some fluctuations at certain iteration counts, the overall trend was one of performance improvement as the number of iterations increased. Specifically, at 32k and 56k iterations, mIoU reached 83.75% and 83.77%, respectively, indicating good convergence. Additionally, the Lite version achieved its best mIoU of 83.96% on OTU_CEUS, slightly higher than the standard version’s 83.32%, further proving the effectiveness of our proposed Lite version. These results not only validate the efficacy of our method but also emphasize the importance of adjusting the model architecture for specific datasets. Moreover, the role of the feature inhibition mechanism in reducing noise and irrelevant features was discussed, contributing to the robustness of the model.

### 5.2 Analysis of lite version superiority over standard version

Notably, during experiments on the OTU_CEUS dataset, we observed that the Lite version outperformed the standard version, which was initially unexpected. Typically, simplifying the model structure can lead to a decrease in performance; however, in this study, the Lite version replaced the complex SMoFFI module with element-wise addition for multi-scale feature fusion, thereby reducing computational load while retaining the advantages of multi-scale feature fusion. Specifically, feature maps *X*_1_ and *X*_3_ were upsampled to the same spatial resolution to ensure compatibility and complementarity of information across different scales. Subsequently, these feature maps were fused into the final fusion feature map *F* = *X*_1_ + *X*_3_ through simple element-wise addition. This simplification unexpectedly led to better performance, possibly due to a better balance between computational complexity and model performance, allowing the Lite version to achieve superior segmentation results while maintaining efficiency. The discussion now includes insights into why this simplified approach might perform better in certain contexts.

### 5.3 Limitations and future work

Despite the promising results achieved by our model, several limitations remain to be addressed. First, the current performance of the model is still somewhat constrained by the limited size and diversity of the available datasets, especially for CEUS modalities in the MMOTU dataset, which may affect its generalization ability. Second, although our Lite version achieves a favorable trade-off between efficiency and performance, there is room for further optimization in terms of architectural design and feature extraction mechanisms. Further analysis of failure cases and their implications for model refinement has been suggested.

Future work will focus on improving the model’s robustness and adaptability through architectural innovations rather than relying solely on increasing data volume. We aim to develop more compact and efficient models that can achieve high performance even with limited training samples. Additionally, we plan to explore self-supervised and semi-supervised learning strategies to reduce the dependency on large-scale labeled data. Through these improvements, we hope to make our method more practical and applicable in real-world clinical settings. We have added plans for validating the model in real-world scenarios and obtaining feedback from radiologists regarding its usability and diagnostic value.

## Data Availability

The data analyzed in this study were obtained from the publicly available MMOTU dataset described in Zhao et al., ‘MMOTU: A Multi-Modality Ovarian Tumor Ultrasound Image Dataset for Unsupervised Cross-Domain Semantic Segmentation’ (https://github.com/cv516Buaa/MMOTU_DS2Net). The data are shared under the terms specified by the authors and can be accessed via the same repository.
